# 

**Published:** 1880-07

**Authors:** 


					﻿Vol. 1.
JULY, 1880.
No. 7.
THE INDEPENDENT
11 r a r 1i11 o n c r;
ft ^OJJTHLY Jo U AL,
DEVOTED TO
MEDICAL, SURGICAL, OBSTETRICAL,
DENTAL and HYGIENICAL
SCIENCE.
EDITED BY
IIARVEY L. BYRD, A. M., M. D.,
AND
BASIL M. WILKERSON, D. D. S., M. D.
“Altera poscit opem res, et con jurat amice.”
BALTIMORE:
THE PRACTITIONER PUBLISHING CO.,
B. M. WILKERSON, Proprietor,
No. 68 North Charles Street.
$2.00 Per Annum in Advance. - - Single Copies, 25 Cents.
X
ENTERED AT THE POSTOFFICE AT BALTIMORE, MI)., AS SECOND-CLASS MATjTER.
				

